# Supplementary material for: l-Ornithine-l-Aspartate (LOLA) Normalizes Metabolic Parameters in Models of Steatosis, Insulin Resistance and Metabolic Syndrome
Source: Pharmaceutics. 2024 Apr 7;16(4):506. doi: 10.3390/pharmaceutics16040506 (PMC11054838; doi:10.3390/pharmaceutics16040506)
Supplement: Supplementary file 1 [file pharmaceutics-16-00506-s001.zip › pharmaceutics-2760487-supplementary.pdf]

Supplementary Materials:

L-Ornithine-L-Aspartate (LOLA) Normalizes Metabolic Parameters in Models of Steatosis, Insulin Resistance and Metabolic Syndrome

Ali Canbay, Oliver Götze, Ozlem Kucukoglu, Sönke Weinert, Roland S. Croner, Theodor Baars, Mustafa K. Özçürümez and Robert K. Gieseler

Table S1. Patient demographics and baseline characteristics.

Age	Sex	Height	Weight	BMI	GPT	GOT	GGT	FFA	M30	M65	CD36	Steatosis	Ballooning	Lobular Inflamm.	Fibrosis	NAS
	1		172.00	46.00	62.0	39.0	50.0		38.72	824.58	9.07	2	2	2	2	6
	1	188.00	152.00	43.01	34.0	23.0	34.0	22.06	118.01	185.51	4.60	3	2	2	2	7
	0	167.00	173.00	62.03	98.0	55.0	114.0	27.40	410.66	564.07	7.23	3	2	3	2	8
	0	172.00	154.00	52.06	.	.	.	22.26	389.29	610.92	18.85	3	2	2	2	7
	1	190.00	142.00	39.34	165.0	120.0	265.0	20.84	511.89	736.87	43.31	3	2	1	2	6
	1	172.00	167.00	56.45	.	.	.	22.06	91.56	406.57	20.59	1	1	2	2	4
36	1	182.00	208.40	62.92	45.0	29.0	25.0	17.76	368.32	416.22	7.80	2	1	2	2	5
29	0	157.00	111.00	45.03	12.0	22.0	10.0	14.84	136.40	253.52	1.99	0	0	1	2	1
43	0	166.00	139.00	50.44	45.0	30.0	17.0	30.34	140.93	248.27	13.57	2	1	2	2	5
26	0	176.00	159.00	51.33	21.0	14.0	41.0		185.45	260.62	4.63	1	1	2	2	4
30	0	159.00	157.00	62.10	31.0	23.0	18.0		141.68	335.02	2.41	1	0	1	2	2
37	0	163.00	104.00	39.14	22.0	23.0	20.0		308.57	471.17	2.81	0	0	1	1c	1
59	1	190.00	188.00	52.08	25.0	16.0	34.0		121.04	402.80	3.82	1	1	2	2	4
47	0	167.00	160.00	57.37	15.0	17.0	77.0		364.41	519.06	6.20	1	1	1	2	3
51	0	171.00	160.00	54.72	34.0	31.0	25.0		648.48	721.48	2.57	2	1	1	2	4
32	F	159.00	139.00	54.98	27.0	22.0	34.0		120.17	260.92	13.80	0	0	1	2	1
52	F	156.00	142.00	58.35	39.0	27.0	64.0		1044.69	1123.67	1.23	2	1	1	2	4
50	F	158.00	124.80	49.99	17.0	19.0	15.0		356.16	491.69	3.12	1	0	1	1a	2
54	M	172.00	114.00	38.53	20.0	27.0	39.0		746.84	909.16	2.78	0	0	1	2	1
36	F	172.00	186.50	63.04	26.0	25.0	27.0		289.63	410.57	2.10	2	1	3	2	6
54	F	164.00	125.50	46.66	13.0	17.0	24.0		402.31	546.67	2.19	1	0	1	1a	2
53	F	177.00	135.40	43.22	21.0	22.0	17.0		60.23	116.61		1	1	1	2	3
53	F	166.00	160.00	58.06	31.0	26.0	63.0		480.26	636.75	4.79	2	1	1	2	4
37	F	165.00	141.00	51.79	27.0	25.0	25.0		308.95	434.34	4.67	1	0	1	2	2
36	F	176.00	135.00	43.58	23.0	21.0	31.0		1071.77	1064.19	2.40	0	0	1	2	1
59	F	167.00	140.00	50.20	13.0	16.0	21.0		418.35	565.50	3.02	1	0	1	1a	2
35	M	170.00	144.00	49.83	34.0	22.0	38.0		180.66	446.11	2.79	1	1	1	2	3
61	F	172.00	167.00	56.45	50.0	32.0	21.0		212.97	315.82		0	0	1	2	1
43	F	183.00	128.00	38.22	20.0	20.0	26.0		177.40	253.26	5.63	1	1	1	2	3
39	F	165.00	144.00	52.89	70.0	49.0	136.0		1154.01	2669.80	0.64	2	1	2	2	5
45	M	180.00	134.00	41.36	50.0	38.0	74.0	25.64	562.10	994.67		2	1	1	2	4
37	F	169.00	132.00	46.22	46.0	58.0	34.0	23.22	674.19	1321.48		3	1	1	2	5
25	F	177.00	212.00	67.67	30.0	26.0	32.0	20.07	132.88	353.16		0	0	1	1c	1
64	F	159.00	150.00	59.33	24.0	25.0	43.0	18.17	221.57	371.00		2	1	1	2	4
36	F	164.00	119.00	44.24	28.0	17.0	29.0	10.69	95.66	316.31		1	0	1	1a	2
63	F	167.00	135.00	48.41	23.0	16.0	16.0	28.99	74.50	346.60		1	0	1	2	2
41	F	166.00	160.00	58.06	12.0	20.0	29.0	18.98	79.31	156.85		1	1	3	2	5
40	M	177.00	152.00	48.52	51.0	35.0	44.0	32.30	289.90	900.34		3	1	1	2	5
24	F	178.00	145.00	45.76	40.0	27.0	55.0	20.50	112.50	455.63		0	0	0 ?	2	0 ?
44	F	178.00	158.00	49.87	8.0	17.0	17.0	28.54	78.89	789.13		1	1	2	2	4
53	M	183.00	185.00	55.24	39.0	32.0	36.0	30.65	161.12	595.21		1	1	3	2	5
42	M	187.00	162.20	46.38	102.0	50.0	52.0		167.05	287.28		2	1	3	2	6
34	F	176.00	142.10	45.87	56.0	52.0	27.0		213.12	516.85		1	1	1	2	3
44	M	189.00	184.00	51.51	14.0	21.0	29.0	23.40	164.49	412.24		0	0	1	2	1
	F	167.00	143.40	51.42	36.0	27.0	25.0		502.51	597.42	26.43	1	1	1	2	3
31	F	180.00	148.00	45.68	24.0	23.0	52.0		290.91	366.95	1.89	2	1	1	2	4
65	F	154.00	137.20	57.85	11.0	17.0	30.0		228.80	334.62	1.71	1	1	3	2	5
44	F	161.00	122.00	47.07	20.0	28.0	20.0		379.11	519.17	3.10	1	1	1	2	3
19	M	172.00	180.00	60.84	48.0	23.0	72.0		365.61	503.05	2.48	2	1	2	1a	5
53	F	175.00	207.00	67.59	36.0	36.0	104.0		292.92	1274.85	9.13	3	1	1	2	5
26	F				18.0	18.0	19.0		219.52	254.22	1.45	0	0	1	2	1
52	F	176.00	134.00	43.26	50.0	45.0	15.0		155.95	455.97	1.73	1	1	2	2	4
44	F	178.00	132.00	41.66	18.0	20.0	18.0		161.15	791.64		1	0	1	1a	2
33	F	173.00	135.00	45.11	56.0	36.0	34.0		351.06	794.70		2	1	2	2	5
45	F	164.00	122.70	45.62	11.0	19.0	38.0					1	1	1	2	3
63	F	160.00	105.00	41.02	32.0	29.0	44.0	23.02	208.93	834.74		1	1	1	2	3
41	F	180.00	145.00	44.75	16.0	16.0	22.0	14.43	128.77	269.78		1	1	1	2	3
36	F	164.00	169.00	62.83	61.0	42.0	30.0	19.54	299.16	573.64		2	1	2	2	5
58	M	191.00	192.00	52.63	38.0	29.0	48.0	18.89	334.42	445.16		1	0	1	2	2
42	F	162.00	135.00	51.44	17.0	20.0	18.0	23.87	305.61	406.79		0	0	1	2	1
41	F	170.00	149.00	51.56	23.0	21.0	27.0	29.38	316.21	745.71		1	1	1	1c	3
50	F	169.00	121.00	42.37	41.0	27.0	24.0	22.35				1	1	1	2	3
43	F	176.00	126.00	40.68	42.0	36.0	20.0	20.83	179.54	499.26		1	1	1	2	3
51	F	165.00	180.00	66.12	18.0	20.0	37.0	24.46	86.22	333.06		2	1	2	2	5
28	F	161.00	118.00	45.52	20.0	15.0	26.0		132.80	371.25		0	0	1	2	1
34	F	155.00	150.00	62.43	18.0	18.0	20.0		122.91	248.26		0	0	1	2	1
32	F	180.00	178.00	54.94	24.0	23.0	47.0					1	2	1	2	4

38	F	161.00	150.00	57.87	18.0	19.0	37.0			1	1	2	2	4
46	F	168.00	153.00	54.21	19.0	22.0	20.0			1	1	1	1a	3
50	M	178.00	220.00	69.44	30.0	26.0	41.0	558.52	980.33	1	1	1	2	3
37	F	158.00	170.00	68.10	39.0	28.0	34.0	112.50	455.63	1	1	1	2	3
24	F	180.00	131.00	40.43	33.0	23.0	28.0	186.36	430.58	1	2	2	2	5
46	F	167	192	68.84	26.0	22.0	20.0	200.44	1013.23	2	2	2	2	6
54	F	156	106	43.56	17.0	21.0	33.0	168.53	507.68	1	1	1	2	3
46	M	176	150	48.42	31.0	30.0	37.0	347.16	822.61	1	1	2	2	4
	F	174	140	46.24	13.0	20.0	17.0			0	0	1	2	1
60	F	154	130	54.82	22.0	21.0	28.0	243.41	316.11	1	1	1	2	3
39	F	173	178	59.47	49.0	34.0	68.0	417.57	696.70	2	1	1	2	4
38	M	165.00	148.00	54.36	36.0	30.0	17.0	221.62		1	1	1	2	3
41	F	176.00	163.00	52.62	13.0	45.0	36.0	269.10	643.03	2	2	3	3	7
37	M	200.00	177.00	44.25	37.0	26.0	23.0	178.12	1350.04	1	0	1	2	2
30	F	171	189	64.64	36.0	34.0	30.0	229.02	620.48	2	1	2	2	5
47	F	170	126	43.60	18.0	22.0	23.0	267.47	301.02	1	1	3	1a	5
46	F	171.00	208.00	71.13	26.0	21.0	24.0	94.51	283.50	1	1	1	2	3
52	F	164.00	157.00	58.37	51.0	31.0	42.0		370.19	2	1	1	2	4
60	F	178	182	57.44	27.0	23.0	30.0			1	1	1	2	3
38	F	160	115	44.92	19.0	22.0	70.0			1	1	1	2	3
47	F	175	143	46.69	31.0	29.0	17.0		186.79	1	1	1	2	3
35	F	162	120	45.72	42.0	29.0	17.0		342.34	1	1	1	2	3
35	F	170.00	161.00	55.71	22.0	40.0	21.0	102.02	216.84	1	0	1	2	2
44	F	174.00	191.00	63.09	22.0	25.0	64.0	196.37	355.07	1	1	2	1a	4
43	F	160.00	142.00	55.47	16.0	19.0	34.0	232.13	240.54	1	1	1	2	3
45	F	168.00	180.00	63.78	15.0	20.0	27.0	380.92	521.25	2	1	1	2	4
56	F							727.72	1462.56	2	1	2	2	5
33	F	175.00	171.00	55.84	47.0	37.0	18.0	266.63	874.81	1	1	1	2	3
29	F	174.00	165.00	54.50	26.0	23.0	21.0	106.70	254.09	1	1	1	2	3
51	F	172.00	125.00	42.25	15.0	20.0	24.0	86.20	176.99	1	1	1	2	3
27	F	170.00	156.00	53.98	20.0	24.0	22.0	326.90	326.72	0	0	1	2	1
39	M	183.00	154.00	45.99	45.0	28.0	67.0	112.07	370.86	2	1	2	1b	5
27	F	169.00	128.00	44.82	27.0	18.0	48.0	366.11	935.15	1	1	1	2	3
40	F	163.00	125.00	47.05	74.0	50.0	132.0		1956.85	3	1	3	1b	7
39	F	178.00	177.00	55.86	47.0	40.0	31.0	315.77	358.49	1	1	1	1a	3
30	F	162.00	142.00	54.11	21.0	22.0	20.0	95.88	1176.46	1	0	1	1b	2
41	F	164.00	173.00	64.32	29.0	26.0	10.0	104.54	680.97	2	1	1	1a	4
44	F	177.00	188.00	60.01	16.0	17.0	33.0	140.54	686.26	1	1	3	1b	5
45	F	167.00	163.00	58.45	27.0	22.0	22.0	325.39	1950.69	2	1	3	2	6
49	M	188.00	206.00	58.28	107.0	69.0	82.0	1105.62	2456.71	3	1	2	2	6
56	F	165.00	161.00	59.14	45.0	33.0	49.0	557.34	763.35	1	1	1	2	3
35	F	172.00	172.00	58.14	15.0	21.0	30.0	126.07	153.32	1	1	1	2	3
51	F	178.00	162.00	51.13	63.0	46.0	45.0	868.67	2755.26	2	1	1	2	4
40	F	175.00	164.00	53.55	18.0	21.0	33.0	120.69	234.44	1	0	1	2	2
22	F	168.00	126.00	44.64	58.0	33.0	41.0	283.73	704.54	2	1	1	2	4
37	F	180.00	154.00	47.53	27.0	26.0	13.0			0	0	1	2	1
30	F	178.00	180.00	56.81	21.0	22.0	20.0			2	1	2	2	5
42	F	169.00	119.00	41.67	20.0	21.0	18.0		234.82	1	1	3	2	5
44	M	180.00	172.00	53.09	48.0	35.0	46.0			3	1	1	2	5
59	F	165.00	172.00	63.18	6.0	21.0	24.0		483.37	1	0	1	2	2
44	F	167.00	113.00	40.52	24.0	21.0	20.0	1309.39	2349.57	2	1	1	2	4
43	F	168.00	156.00	55.27	41.0	27.0	25.0	1182.89	1339.92	1	0	1	2	2
28	M	175.00	147.00	48.00	53.0	32.0	28.0	947.62	2971.51	2	2	2	2	6
38	F	168.00	158.00	55.98	30.0	29.0	40.0			1	1	1	1a	3
60	F	162.00	118.00	44.96	23.0	24.0	60.0			1	1	1	2	3
34	F	165.00	165.00	60.61	8.0	21.0	16.0			1	0	1	2	2
53	F	168.00	125.00	44.29	33.0	22.0	31.0			3	1	2	2	6
52	F	165.00	109.00	40.04	44.0	31.0	56.0			3	1	3	2	7
27	F	195.00	260.00	68.38	8.0	16.0	51.0			1	0	2	2	3
31	F	160.00	107.00	41.80	38.0	31.0	25.0			1	0	1	2	2
51	F	158.00	130.00	52.07	51.0	41.0	42.0			2	1	3	2	6
35	F	165.00	111.00	40.77	111.0	70.0	112.0			2	1	2	2	5
31	F	163.00	170.00	63.98	28.0	25.0	30.0	269.80	416.55	1	0	1	2	2
45	F	170.00	200.00	69.20	22.0	23.0	23.0	180.39	438.01	1	1	2	2	4
50	M	181.00	126.00	38.46	49.0	42.0	86.0	441.37	956.84	2	1	2	2	5
39	M	194.00	248.00	65.89	34.0	23.0	36.0	103.85	319.56	2	1	3	2	6
36	F	173.00	143.00	47.78	28.0	19.0	21.0	145.93	245.26	1	0	2	2	3
45	F	168.00	130.00	46.06	33.0	25.0	39.0	218.24	436.07	1	0	1	2	2
37	F		117	49	15.0	17.0	20.0			0	0	1	1a	1
50	F		157	50	27.0	22.0	96.0			1	0	1	2	2
46	F		174	59.5	18.0	20.0	20.0			1	1	1	2	3
30	F		106	37.5	41.0	65.0	27.0			2	1	1	2	4
43	F		222	79	16.0	21.0	21.0			1	0	2	1a	3
56	F		188.4	65	24.0	23.0	21.0	138.58	170.75	1	2	2	2	5
41	F		123	43	27.0	21.0	34.0	869.55	1275.66	2	2	2	2	6
38	F		128	43.5	34.0	28.0	22.0	110.80	110.89	1	1	1	0	3
43	F		186.8	63	30.0	28.0	14.0	144.12	229.66	2	1	1	2	4
39	F		166	56	18.0	24.0	37.0	425.67	330.96	2	1	0	2	3
52	F		123	44	32.0	59.0	226.0	569.85	849.16	2	2	1	4	5
38	F		147.6	52	24.0	17.0	13.0	284.90	1494.25	3	2	2	1c	7
53	F	163	114.2	43.0	31.0	24.0	71.0			3	2	1	2	6
40	M	185	186	54.0	86.0	50.0	28.0			1	1	0	2	2
58	F	178	142	45.0	28.0	22.0	27.0			2	2	0	1C	4
30	F	163	147	55.0	14.0	15.0	13.0			0	0	1	1C	1
61	F	168	148	52.0	15.0	23.0	46.0			0	1	0	2	1
43	M	181	141	43.0	27.0	20.0	30.0			1	1	0	3	2
41	M	170	140	48.0	78.0	41.0	79.0			3	2	1	2	6
47	F			47.0	14.0	20.0	13.0			0	2	2	2	4
60	F	177	137	44.0	17.0	24.0	24.0			1	2	2	2	5

27	F	177	153	48.0	62.0	55.0	47.0	3	0	1	2	4
54	M	186	196	56.0	42.0	29.0	36.0	3	1	0	2	4
34		176	152	49.0	141.0	92.0	236.0	3	1	1	3	5
51	M	174	144.00	46.00	33.00	23.00	37.00	3	2	3	3	8
50	F	178	197	62	19	/	35	2	1	1	2	4
31	M	189	169	47	48	23	35	2	2	1	2	5
43	F	167	148	53	20	/	16	3	2	2	2	7
38	F	165	156	57	12	/	16					
51	F	168	148	52	39	32	47	1	2	2	3	5
25	M	/	/	/	30	/	72	0	0	1	3	1
50	F	168	111.6	39.5	101	50	99	0	1	1	2	2
54	F	160	158	62	27	/	32	0	2	1	2	3
33	F	169	106.8	37.4	51	23	83	2	2	0	3	4
34	F	169	118	41	15	/	14	0	0	0	2	0
50	M	195	179	47	31	/	21	0	0	1	2	1
41	F	172	150	50.7	36	32	13	0	0	0	1C	0
38	F	170	121	42	34	/	16	0	2	1	3	3
46	F	168	123	43.6	124	102	165	2	2	3	3	7
51	F	185	147	43	24	/	13	1	2	1	3	4
63	F	171	140	47.8	18	/	28	1	1	1	1A	3
62	F	165	135	49.6	19	/	31	3	2	1	3	6
49	F	168	112	40	18	/	21	1	1	2	1C	4
44	M	180	206.6	63.8	36	/	33	2	1	2	4	5
45	F	171	145.3	49.7	29	/	15	1	1	1	2	3
42	F	175	180	58.9	37	33	47	1	1	2	2	4
40	F	170	157.7	54.6	18	/	31	1	2	2	2	5
27	F	172	149.2	50.4	45	29	35	3	2	2	1A	7
50	F	172	130	45	36	27	74	3	2	3	1C	8
25	F	173	162.3	54.2	23	/	32	0	1	2	2	3
43	F	166	143.5	43.7	21	/	10	0	1	1	1C	2
44	M	183	146	42.7	8	/	15	1	2	1	2	4
53	F	170	128	44.5	15	/	18	0	1	1	1C	2
50	F	166	123	44.7	35	/	61	0	1	1	2	2
34	F	166	166	59	21	/	13	3	2	3	2	8
53	M	185	155	45.3	120	79	109	3	1	2	2	6
44	F	170	153	52.9	24	/	14	1	2	2	2	5

Table S2. Antigens, primary anti-human antibodies (top) and secondary conjugated antibodies (bottom). Target antigens are listed in alphabetical order (except for Greek letters).

1. Primary Anti-Human Antibodies

Antigen	Type	Host	Clone	Class	Conjugate	IB	IHC	Context	Vendor	Catalog No.
ACC1*	mAb	R	C83B10	IgG	–	+	–	Gluconeogenesis + *de-novo* lipogenesis	CS	3676
β-Actin	mAb	M	AC-15	IgG	–	+	–	Microfilament protein	SA	A1978
AIF	mAb	R	D39D2	IgG	–	+	–	Apoptosis-inducing factor + caspase-independent apoptosis	CS	5318
AKT	pAb	R	–	IgG	–	+	–	Gluconeogenesis	CS	9272
AMPK-α	mAb	R	D5A2	IgG	–	+	–	Master regulator of energy metabolism, homeostasis, lipogen-esis, gluconeogenesis	CS	5831
Arginase1	mAb	R	D4E3M	IgG	–		+	Urea cycle		
Bax	pAb	R	–	IgG	–	+	–	Apoptosis through mitochondrial stress	CS	2772
BCAT2	mAb	R	D8K3O	IgG	–	+	–	BCAA metabolism	CS	79764
β-Catenin	mAb	R	D10A8	IgG	–	+	–	EMT/WNT signaling	CS	8480
CD36	pAb	R	–	IgG	–	+	–	Fatty acid transporter	NB	NB400-144
CD73	mAb	R	D7F9A	IgG	–	+	–	AMP to adenosine-converting enzyme	CS	13160
Citrate synthase	mAb	R	D7V8B	IgG	–	+	–	Mitochondrial enzyme (TCA cycle)	CS	14309
Cleaved caspase 3	pAb	R	–	IgG	–	+	–	Apoptosis	CS	9661
CPS1	pAb	R	–	IgG	–	+	+	Urea cycle	NB	NBP1-86019

E-Cadherin	mAb	M	36/E	IgG	–	+	–	Carcinogenesis (EMT) and zonation	BD	610182
FASN	mAb	R	C20G5	IgG	–	+	–	*De-novo* lipogenesis	CS	3180
GAPDH	mAb	R	14C10	IgG	–	+	–	Energy metabolism	CS	2118
GLUL	mAb	R	D2O3F	IgG	–	+		NH_3 homeostasis	CS	80636
HSP27	mAb	M	G31	IgG_1	–	+	–	Mainly cytosolic, but also in other organel-les, except mitochon-dria; inhibits apoptosis, among other functions	CS	2402
HSP60	mAb	R	D6F1	IgG	–	+	–	Mitochondrial protein import and macro-molecular assembly	CS	12165
OTC	pAb	R	–	IgG	–	+	+	Urea cycle	NB	NBP1-87408
p21	mAb	R	12D1	IgG	–	+	–	Tumor suppressor, inhibitor of cell cycle progression	CS	2947
p-AKT	mAb	R	193H12	IgG	–	+	–	Gluconeogenesis	CS	4058
p-AMPK-α	mAb	R	40H9	IgG	–		–	Master regulator of energy metabolism, homeostasis, lipogen-esis, gluconeogenesis	CS	2535
PARP	pAb	R	–	IgG	–	+	–	Enzyme involved in transcriptional regulat-ion, DNA repair, cell death, inflammation	CS	9542
PHB1	pAb	R	–	IgG	–	+	–	Mitochondrial chaper-one acting as a tumor suppressor, and invol-ved in homeostasis, cell cycle control, proliferation, and apoptosis	CS	2426
PPAR-γ	mAb	R	C26H12	IgG	–	+	–	*De-novo* lipogenesis	CS	2435
p-PTEN	mAb	R	44A7	IgG	–	+	–	Tumor suppressor	CS	9549
PTEN	mAb	R	138G6	IgG	–	+	–	Tumor suppressor	CS	9559
Slug-Snail	pAb	R	–	IgG	–	+	–	Carcinogenesis (EMT)	abcam	Ab180714
SOD1	mAb	M	71G8	IgG	–	+	–	Antioxidant	CS	4266

2. Secondary Conjugated Antibodies

**Antigen**	**Type**	**Host**	**Clone**	**Class**	**Conjugate**	**ICC**	**IHC**	**Application**	**Vendor**	**Catalog No.**
Mouse IgG	N/A	H	N/A	N/A	HRP	+	+	Visualizing bound primary murine IgG	CS	7076
Rabbit IgG	N/A	H	N/A	N/A	HRP	+	+	Visualizing bound primary rabbit IgG	CS	7074

* Abbreviations: ACC1, acetyl-CoA carboxylase 1; AIF, apoptosis-inducing factor; AKT, PI-3'K, phosphoinositide 3-kinase; AMPK-α, AMP-activated protein kinase α; Bax, BCL2-associated X protein; BCAT2, branched-chain amino acid aminotransferase 2; BD, Becton Dickinson, Heidelberg, Germany; CD36/FAT, fatty acid translocase; CD73, ecto-5′-nucleotidase (AMP-to-adenosine converting enzyme); CS, Cell Signaling Technology Europe, Frankfurt/Main, Germany; CPS1, carbamoyl phosphate synthetase 1; EMT, epithelial-mesenchymal transition; FASN, fatty acid synthase; GAPDH, glyceraldehyde-3-phosphate dehydrogenase; GLUL, glutamate-ammonia ligase (obsolete: glutamine synthetase); H, horse; HRP, horseradish peroxidase; HSP27, heat shock protein 27; HSP60, heat shock protein 60; IB, immunoblotting; IHC, immunohistochemistry; M, mouse; mAb, monoclonal antibody; N/A, not available; NB, Novus Biologicals, Centennial, CO, USA via Bio-Techne, Wiesbaden-Nordenstadt, Germany; OTC, ornithine transcarbamylase; p21, a tumor suppressor; pAb, polyclonal antibody; pAKT, phosphorylated AKT; pAMPK-α/AMPK-α, ratio of phosphorylated AMPK-α to non-phosphorylated AMPK-α; PARP, poly ADP ribose polymerase; PPARα, peroxisome proliferator-activated receptor α; p-PTEN,

phosphorylated phosphatase and tensin homolog; R, rabbit, SA, Sigma-Aldrich (formerly) / now: Merck, Darmstadt, Germany; Slug, a key EMT regulator; Snail 2, a key EMT regulator; SOD1, superoxide dismutase 1;TCA, tricarboxylic acid.

Table S3. Primer oligonucleotide sequences for qRT-PCR in alphabetical order of their transcript-related specificities. Transcripts are listed in alphabetical order. See footer for abbreviations.

Transcript	Primer Sequence	Context	Vendor	Catalog No.
bcat2	Not available	BCAA metabolism	Q	QT00077406
bckdha	Not available	BCAA metabolism	Q	QT00057988
bckdk	Not available	BCAA metabolism	Q	QT00088389
cd36	Forward: 5′-AACAGTTCTCTCAAAGTTCTGACTTG-3′<br>Reverse: 5′-AACAGAGGCTGACAACTTCACA-3′	Fatty acid transporter	EG	N/A
cd73	Forward: 5′-CTCCTCTCAATCATGCCGCT-3′<br>Reverse: 5′-CAAATGTGCCTCCAAAGGGC-3′	Carcinogenesis (EMT) and zonation	EG	N/A
cpt1	Forward: 5′-TGTGCTGGATGGTGTCTGTC-3′<br>Reverse: 5′-CAAACTGGACCGGGAGGAAA-3′	Energy metabolism	EG	N/A
fasn	Forward: 5′-GATGCGGGAATACAGGTGAC-3′<br>Reverse: 5′-GACACTGCCGTGGAGAACAT-3′	*De-novo* lipogenesis	EG	N/A
foxo1	Forward: 5'-TTATGACCGAACAGGATGATCTTG-3'<br>Reverse: 5'-TGTTGGTGATGAGAGAAGGTTGAG-3'	Insulin-dependent gluconeogenesis	EG	N/A
gadph	Forward: 5'-GGTGACCAGGCGCCCAATACG-3'<br>Reverse: 5'-TTCTTTTGCGTCGCCAGCCGA-3'	Household enzyme and, *inter alia*, energy metabolism, glycolysis enzyme, transcription activation, and initiation of apoptosis	EG	N/A
hnf4	Forward: 5'-CGCACTGCCGGCTAAATCTGC-3'<br>Reverse: 5'-CGACACGTCCCCATCAGAAGGC-3'		EG	N/A
hprt	Forward: 5′GACCAGTCAACAGGGGACAT-3′<br>Reverse: 5′-CTTGCGACCTTGACCATCTT-3′	Household enzyme	EG	N/A
ikk-β	Forward: 5′-TGTCACAGGGTAGGTGTGGA-3′<br>Reverse: 5′-AAGGCCCTGGATGACATCTT-3′	Inflammation	EG	N/A
il-6	Forward: 5'-TTTCACCAGGCAAGTCTCCT-3'<br>Reverse: 5'-AGGCACTGGCAGAAAACAAC-3'	Inflammation	EG	N/A
p21^{Cip1}	Forward: 5'-GACTCTCAGGGTCGAAAACG-3'<br>Reverse: 5'-GGATTAGGGCTTCCTCTTGG-3'	Tumor suppressor, inhibitor of cell cycle progression	EG	N/A
p53	Forward: 5'-GCGCACAGAGGAAGAGAATCTCCG-3'<br>Reverse: 5'-TTTGGCTGGGGAGAGGAGCTG-3'	Tumor suppressor, involved in carcinogenesis (EMT) and zonation	EG	N/A
pgcα	Forward: 5′-CAGCACACTGCTGCTCGTAATA-3′<br>Reverse: 5′-AGCTTTGCCTACATGGCAGC-3′	Energy metabolism, insulin-dependent gluconeogenesis, fatty acid β-oxidation	EG	N/A
pparα	Forward: 5'-ACTCCACCTGCAGAGCAACCA-3'<br>Reverse: 5'-TAGATCTCCTGCAGTAGCGGG-3'	Lipogenesis, fatty acid β-oxidation	EG	N/A
pten	Forward: 5'-GAACTGGCAGGTAGAAGGCA-3'<br>Reverse: 5'-CATAACGATGGCTGTGGTTG-3'	Carcinogenesis (EMT) and zonation	EG	N/A
scd	Not available	*De-novo* lipogenesis	Q	QT00052381
sdha	Forward: 5′CAAAGGGCTTCTTCTGTTGC-3′<br>Reverse: 5′-ACACAGACCTGGTGGAGACC-3′	Participates in TCA cycle and electron transport chain (household enzyme)	EG	N/A

sirt1	Forward: 5′-AACGATTTGGTGGCAAAAAC-3′<br>Reverse: 5′-CAGTGGCTGGAACAGTGAGA-3′	*De-novo* lipogenesis, fatty acid β-oxidation, insulin-dependent gluconeogenesis	EG	N/A
smile	Forward: 5′-AAAAGAGGCGGAGAAAGTCC-3′<br>Reverse: 5′-CTCTGAAGAGCGAGGTGGTC-3′	*De-novo* lipogenesis	EG	N/A
srebp-1c	Forward: 5'-CTGACCGACATCGAAGGTGA-3′<br>Reverse: 5'-GAAAGTGCAATCCATGGCTCC-3	*De-novo* lipogenesis, gluconeogenesis	EG	N/A
tnf-α	Forward: 5'-GAGCTCCACGCCATTGGCCA-3'<br>Reverse: 5'-GCCCCCAGAGGGAAGAGTTCCC-3'	Inflammation	EG	N/A

* Abbreviations: BCAT2, branched-chain amino acid aminotransferase 2; BCKDHA, branched-chain keto acid dehydrogenase; BCKDK, branched-chain keto acid dehydrogenase kinase; CD36, fatty acid translocase; CD73, ecto-5′-nucleotidase (AMP-to-adenosine converting enzyme); CPT1, carnitine palmitoyltransferase 1; EG, Eurofins Genomics, Ebersberg, Germany; FASN, fatty acid synthase; FOXO1, forkhead box protein O1; GAPDH, glyceraldehyde-3-phosphate dehydrogenase; HNF4, hepatocyte nuclear factor 4; HPRT, hypoxanthine phosphoribosyl transferase 1; IKK-*β*, I*κ*B kinase-*β* (part of the IKK kinase complex, i.e., the core element of the NF-κB cascade); IL-6, interleukin 6, p21Cip1, a tumor suppressor; p53, a tumor suppressor; PGC1*α*, peroxisome proliferator-activated receptor γ coactivator 1-*α*; PPAR*α*, peroxisome proliferator-activated receptor *α*; PTEN, phosphatase and tensin homolog; Q, Qiagen, Hilden, Germany. SCD1, stearoyl-CoA desaturase; SDHA, succinate dehydrogenase complex, subunit A; SIRT1, for short: sirtuin 1 (sirtuin silent information regulator 1); SMILE, small heterodimer partner interacting leucine zipper protein; srbf1, besides other isotypes also encoding for the hepatic isoform, SREBP-1c, sterol regulatory element-binding protein-1c; TNF-*α*, tumor necrosis factor *α*; TCA, tricarboxylic acid.
